# Health effects of long-term care insurance on spouses of disabled people: a quasi-experimental study

**DOI:** 10.1186/s12877-023-04344-9

**Published:** 2023-10-19

**Authors:** Yanling Yi, Jing Xin, Junxia Liu, Jing Wu

**Affiliations:** 1 School of Public Administration, Zhongnan University of Economics and Law, 182 Nanhu Road, Guanshan Street, Hongshan District, Wuhan, China; 2 School of Public Administration, Yanshan University, Qinhuangdao, China

**Keywords:** Long-term care insurance, Health effects, Pain, Self-reported health, Depression, China

## Abstract

**Background:**

It is still uncertain whether and how formal long-term care (LTC) systems affect the health status of family members. This paper examines the health effects of long-term care insurance (LTCI) on spouses of disabled people in China.

**Methods:**

The data is from China Health and Retirement Longitudinal Survey (CHARLS), a longitudinal survey of a nationally representative sample of Chinese residents aged 45 or older and their spouses, and China City Statistical Yearbook. Exploiting the regional variation in the implementation of LTCI in the first round of pilot cities in China, a difference-in-difference (DID) strategy is applied to identify the causal effects of LTCI on the health status of spouses of disabled people. We carefully identify the causal effects by controlling for city-level covariates, testing common trends between the treatment and control groups, combining propensity score matching (PSM) with DID, selecting the second round of pilot cities as the control group, controlling for city fixed effects (FE) instead of individual FE, and evaluating selection bias from omitted observable and unobservable factors.

**Results:**

The introduction of LTCI in China reduces the number of painful body parts and the self-reported health score significantly, indicating that spouses of disabled people get physical health benefits from LTCI coverage. However, the impact of LTCI on the depression index remains ambiguous and needs to be analyzed further. LTCI improves the physical health status of spouses of disabled individuals mainly through the time reallocation channel, while the impact of the consumption promotion channel has not been verified. Furthermore, the beneficial effects of LTCI on physical health are stronger for spouse caregivers and spouses with lower-level education and lower household income.

**Conclusion:**

These findings demonstrate that LTCI not only improves the health status of family caregivers by reducing their caregiving burden but also has beneficial health effects on non-caregiver family members. Policy designs of LTCI should emphasize the orientation of home and community-based care services (HCBS), which can not only satisfy the care preferences of disabled individuals, reduce the care burden on family caregivers, promote the health of all family members, but also prevent a large number of disabled individuals from choosing high-cost institutional care and reduce the financial burden of the LTCI Fund.

**Supplementary Information:**

The online version contains supplementary material available at 10.1186/s12877-023-04344-9.

## Background

To cope with the growing need for LTC services, which is argued to be mainly driven by the increasing aging population, many countries chose to establish public LTC systems. Unlike those providing formal LTC services under universal or selective public LTC programs such as Nordic countries (Norway, Sweden, Denmark, and Finland), the UK, USA, Spain, etc., which rely on general taxation as the prime source of funding, China introduced LTCI program basing on a social insurance system following Germany, the Netherlands, Japan, Korea and so on. Despite its relatively short history, LTCI has been developing rapidly in China. The Chinese government launched the LTCI pilot officially in 15 cities in 2016 and expanded this program to another 14 cities in 2020. In 2021, it covered 144.607 million people in all 49 pilot cities, and there were 1.087 million disabled people who received LTCI benefits.

Formal LTC systems are not only demonstrated to be beneficial to care recipients directly but also illustrated to have spillover effects on their family members, especially on family caregivers [1–3]. However, the impacts of Formal LTC systems on both caregiving and financial burdens of households, and the health status of family members have so far remained uncertain. In Canada, an increase in the generosity of publicly financed home care induces households to choose formal care over informal care [4]. Although no evidence to illustrate the effect of LTCI on the substitution effect of formal LTC for informal care at the extensive margin, at the intensive margin there is statistically significant evidence in South Korea [5]. Japan’s LTCI reform in 2006, which decreased upper limits of LTCI coverage and limited types and frequencies of available home help or daycare services for non-institutionalized older persons with low care needs, has finally aggravated caregiving burdens and deteriorated health outcomes of family caregivers [3]. However, another study on the effects of LTCI in Japan reports that although the introduction of LTCI reduces the proportion of household expenditure paid for formal LTC by 5% and the caring hours from family by 0.81 h per day, there are no effects on the subjective health status of caregivers [6]. Even more, Carrino et al. [7] find that an increase in formal care utilization would improve both the probability of informal care use and the hours of informal care received significantly, conditional on receiving informal care, implying that Formal LTC systems may aggravate the burden of family members.

The LTCI pilots in China add new experiences and evidence of the effects of formal LTC systems, which would be more helpful to many middle-income and developing countries that also have been facing increasing demands for LTC services and considering establishing financing systems for them. However, rigorous evaluations of the effects of LTCI pilots in China are far from enough. Although several studies examine the impacts of LTCI pilots in China on medical expenses, hours of informal care, and the well-being of older adults [8–12], the spillover effects on family members haven’t received enough attention. One recent study investigates the impact of LTCI pilots on female labor supply in rural China [13], but the health effects on family members of frail individuals are still unknown. Exploiting the regional variation of LTCI pilots in China, we sought to address the following questions. First, does the introduction of LTCI affect the health status of spouses of disabled people? Second, if there are health effects of LTCI on spouses of disabled people, what are the possible channels? Third, are there heterogeneous health effects across different individual characteristics?

The DID strategy is applied to examine the health effects of LTCI on spouses of disabled people using nationally representative survey data from CHARLS and statistical data from China City Statistical Yearbook. We first illustrate that LTCI reduces the number of painful body parts and the self-reported health score significantly, indicating that LTCI has improved the physical health status of spouses of disabled people. Then we demonstrate that LTCI exerts health effects mainly through the time reallocation channel because LTCI reduces the probability of spouse caregiving, and increases spouses’ actual sleep hours at night, but hasn’t significant impacts on households’ consumption. Furthermore, we examine heterogeneous effects of LTCI across individual characteristics and caregiving status and find that the health effects are larger for spouses taking care of his/her disabled partner, with lower-level education or lower household income.

This study makes several contributions to the literature as follows. Firstly, we provide empirical evidence of the health effects of LTCI on family members of disabled people, which has not been rigorously examined to the best of our knowledge. Secondly, we carefully identify the causal effects between the implementation of LTCI and the health status of family members of disabled people by controlling for city-level covariates, testing common trends between the treatment and control groups, combining PSM with DID, selecting the second round of pilot cities as the control group, controlling for city FE instead of individual FE, and excluding the overwhelming impact from omitted observable and unobservable factors. Thirdly, we also demonstrate that the introduction of LTCI in China significantly alleviates the care burden of families with disabled individuals, and will be beneficial to the physical health status of family members by increasing their time invested in health production. Fourthly, we argue that LTCI is more helpful to family members taking care of disabled individuals and those with disadvantaged socio-economic status in dealing with the financial and caregiving challenges from LTC needs.

## Methods

### Institutional settings

In response to the ever-increasing need for LTC services, the Chinese government declared to start LTCI in 15 cities in July 2016, while Jilin and Shandong provinces, as key pilot provinces, could select some other cities that were not on the list to implement LTCI as well. Actually, before the official announcement, three of the 15 pilot cities, namely Qingdao, Changchun, and Nantong, had implemented LTCI in July 2012, May 2015, and January 2016 respectively. Local governments of pilot cities were entitled to design differential rules according to their actual conditions under the guidelines from the central government. Therefore, arrangements of LTCI programs vary across these pilot cities.

In brief, most of the pilot cities implement LTCI in 2016 and 2017 while 2 cities started their programs before that period; most LTCI programs of the pilot cities cover only urban employees and retirees enrolled in the urban employee basic medical insurance (UEBMI) while some other cities expand their LTCI coverages to urban and even rural residents enrolled in the urban and rural residents basic medical insurance (URRBMI); all these programs are financed mainly by public medical insurance funds and most of the pilot cities also raise money from various channels, such as governments’ subsidies, employers’ or individuals’ contributions, or social donations; in most of these cities, only individuals with severe disability are qualified to receive LTCI benefits while some other cities offer payments to people with moderate disability, mild disability or severe dementia; most of the pilot cities cover service benefits only while some cities also compensate family caregivers with caregiving allowances. Detailed information on the key policy issues of LTCI programs in 15 pilot cities is summarized in Supplementary Table 1.

### Data source

All four waves of survey data from CHARLS and the statistical data from China City Statistical Yearbook published in corresponding years are used in this study. CHARLS is a longitudinal survey that aims to collect high-quality microdata of a nationally representative sample of Chinese residents aged 45 or older and their spouses, including a wide range of information on demographic characteristics, family structure, health status, work or retirement, household income and consumption and so on. The respondents chosen through multistage probability sampling of four stages, namely county, communities (in urban areas) or villages (in rural areas), households, and individuals in the national baseline survey in 2011, were to be followed about every two or three years. The final individual participants are from 450 communities (or villages), 150 counties, and 28 provincial administrative regions in China. Until now, CHARLS has finished four waves of surveys in 2011, 2013, 2015, and 2018 respectively, with a final sample of 19,000 respondents from 12,400 households. China City Statistical Yearbook contains main statistical data on the socio-economic development of more than 650 cities across the country, providing information on population, economy, health conditions, etc. for this study to control for city-level factors.

### Study sample

**Fig. 1:**
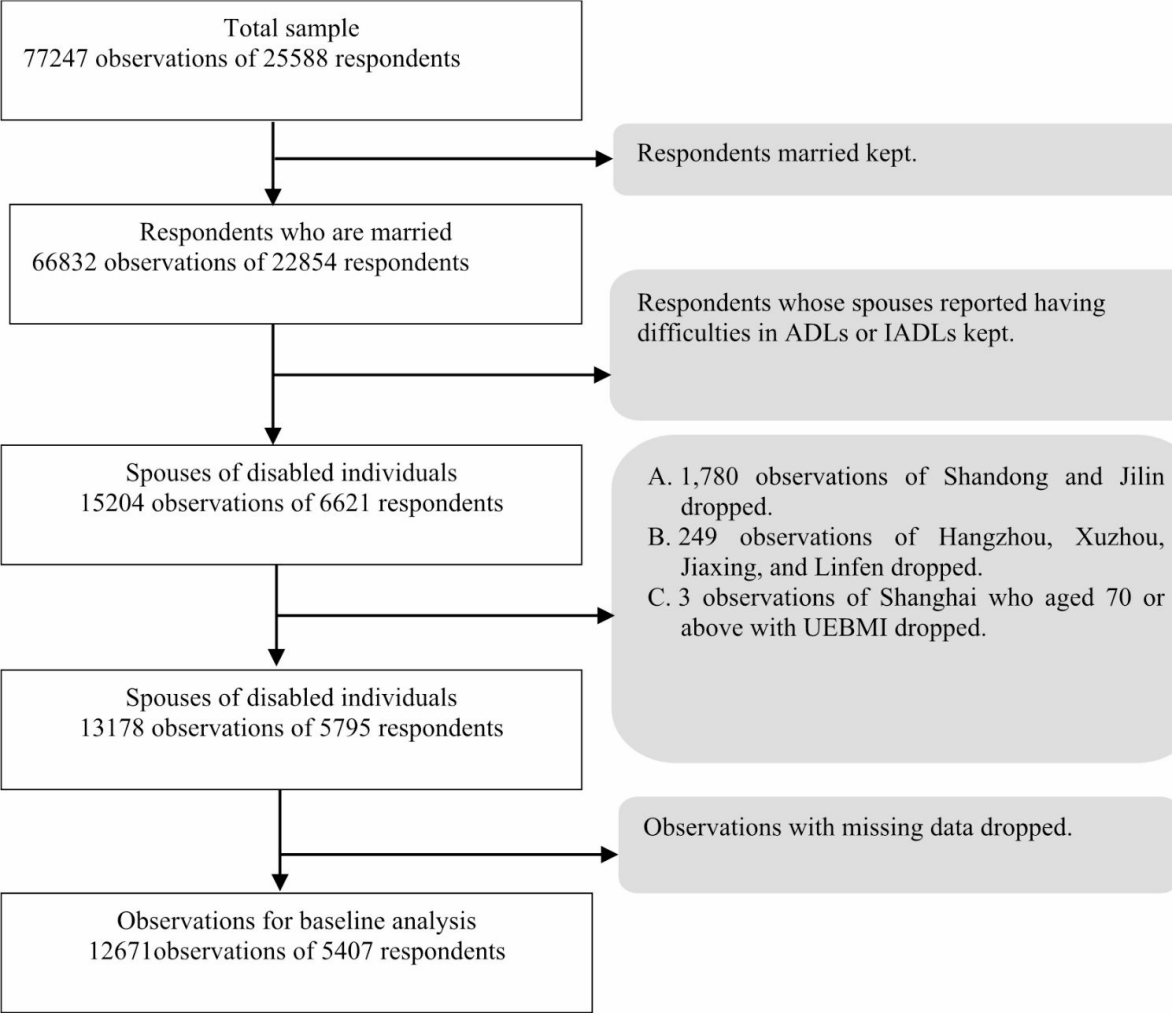
The process of sample selection

Among a total sample of 77,247 observations, 12,671 ones finally remained. Figure 1 shows the process of sample selection. Disabled people are defined as individuals who reported having difficulties in activities of daily living (ADLs) or instrumental activities of daily living (IADLs) [14]. The exclusion of observations in the Shandong and Jilin provinces is to address the endogeneity from the implementation of LTCI within the two key pilot provinces. The elimination of observations in the four autonomous pilot cities is to avoid interferences from autonomous pilot cities. And because elders aged 70 or above with UEBMI in Shanghai were covered by the medical care plan for the oldest old implemented in Shanghai in 2015, their observations are also excluded.

### Outcome variables

Consistent with previous studies [3, 4, 14–16], three outcome variables, including body pain, self-reported health, and depression are selected to measure the health status of spouses of disabled people from multiple aspects. Body pain is measured by the number of painful body parts, assessed with the question “Please list all parts of the body you are currently feeling pain.” The possible answers contain the 15 parts of a human body such as the head, shoulder, arm, etc., and an option of “other”. Therefore, the variable ranges from 0 to 16. Self-reported health is scored as 1 to 5 representing the best to the poorest health status respectively, according to the question “Would you say your health is very good, good, fair, poor or very poor?” For depression, there are 10 items of CESD-10, a simplified form of the Center for Epidemiologic Studies Depression Scale (CES-D) with equal predictive accuracy [17] in the questionnaire of CHARLS, which enquire about the frequencies of 8 negative and 2 positive emotions during the last week, leading to a total score ranging from 1 to 30, with higher scores representing greater symptoms of depression.

### Key independent variable

The key independent variable is whether the city in which the disabled individual lives implemented LTCI during 2016 and 2017. As we aim to investigate the health effects of LTCI in 11 pilot cities, namely Chengde, Qiqihaer, Shanghai, Suzhou, Ningbo, Anqing, Shangrao, Jinmen, Guangzhou, Chongqing, and Chengdu, spouses are classified into the treatment group if their disabled partners dwelled in these cities, on the contrary, those in other cities fell into the control group.

### Empirical strategies

According to the analysis of Grossman, health is creative of utilities to consumers not only as a consumption commodity but also as an investment commodity. Consumers can increase the stock of health in the (*i* + 1)th period by producing investment in health capital during the *i*th period, and at the same time, consumers are constrained by both budget and time [18]. LTCI would affect the health of family members of disabled people by loosening both budget and time constraints. By reducing current and expected future financial burdens, and further increasing households’ consumption of daily necessities, LTCI may be beneficial to the health of spouses of disabled people; while devoting the saved time from changing households’ caregiving decisions and decreasing caregiving hours of spouses to health production, LTCI could also improve the health status of spouses of disabled people. Therefore, LTCI can influence all households in the pilot cities, whether they are eligible for the benefits and enrolled in the program or not, making intent-to-treat effects, rather than treated effects, more attractive in this study, just as in studies of Angelucci and Giorgi [19] and Huang and Zhang [20].

The standard DID is applied to identify the causal effect of LTCI on the health status of spouses of disabled people because among the 12 pilot cities covered by CHARLS, 11 officially implemented LTCI in 2016 and 2017, and the four waves of data in 2011, 2013, 2015 and 2018 cover three waves before and one wave after the official implementation of LTCI, making the standard DID method possible. Qingdao implemented LTCI early in 2012, resulting in three waves of data after the event. However, only one pilot city is not representative, contributing to the fact that the staggered DID has no strengths to the standard DID. Meanwhile, four waves of panel data make the control for two-way FE possible. Therefore, the equation is as follows:

1 $${Health}_{ict}={\beta }_{0}+{\beta }_{1}{Treat}_{ic}\times {Post}_{t}+{\beta }_{2}{X}_{ict}+{\beta }_{3}{W}_{ct}+{\tau }_{t}+{\alpha }_{i}+{\epsilon }_{ict}$$

The subscripts *i, c*, and *t* represent individuals, cities, and years respectively. The dependent variable *health*_*ict*_ is the potential health outcomes of a disabled individual’s spouse *i* living in city *c* in year *t*, including the number of painful body parts, self-reported health score, and depression index.

The key independent variable is *Treat*_*ic*_*×Post*_*t*_, where *Post*_*t*_ is a binary variable that equals 1 for 2018 and 0 for 2011, 2013, and 2015, and *Treat*_*ic*_ is a dummy variable with a value of 1 for the treatment group and 0 for the control group.

*X*_*ict*_ is a vector of time-variant individual characteristics, and *Wct* is a vector of variables that measure the time-varying characteristics at the city level. While *τ*_*t*_ captures time FE, *α*_*i*_ represents individual FE, and *ε*_*ict*_ is the random error term. We cluster standard errors at the city level to overcome the possible correlations among disturbance terms of different individuals in the same city.

Our main interest is *β*_*1*_, which estimates intent-to-treat effects averaging across all spouses of disabled people in the pilot cities regardless of whether the disabled individuals are enrolled or not enrolled in the programs, eligible or ineligible for the benefits. The strategy is consistent with previous studies of Angelucci and Giorgi [19], who find that the ineligible households in the same village could also benefit from the cash transfer program indirectly, and Huang and Zhang [20], who demonstrate that the New Rural Pension Scheme in China not only has significant effects on the age-eligible group in the rural area irrespective of whether they receive the pensions or not but also reduces the proportion of farm-work and increases the proportion of nonfarm-work of the age-ineligible group with rural hukou.

According to the parallel trend assumption, the key assumption of DID, the potential trend of outcomes in the treatment group would be parallel to that in the control group without the implementation of LTCI, conditional on the covariates of Eq. (1). The event-study specification is applied to test the assumption. The equation is as follows.

2 $${ Health}_{ict}={\beta }_{0}+\sum _{t=2011}^{2013}{\beta }_{t}{Treat}_{ic}\times {Post}_{t}+{{\beta }_{2018}Treat}_{ic}\times {Post}_{t}+{\beta }_{2}{X}_{ict}+{ \beta }_{3}{W}_{ct}+{\tau }_{t}+{\alpha }_{i}+{\epsilon }_{ict}$$

*β*_*t*_ represents the estimates of 2011 and 2013 respectively, and 2015 is the benchmark year.

One concern is that the selection of pilot cities of LTCI may not be random. Actually, it was central or local governments that determined whether a city could be selected for the pilot, probably depending on the preexisting city-level economic, social, and cultural characteristics. To address this concern, based on the controlling for two-way FE to eliminate the biases from all time-invariant observable and unobservable factors, five city-level variables are added in the regressions as specified in Eq. (1) to alleviate biases caused by time-variant city-level factors. Furthermore, we combine PSM and DID to provide a more similar control group because there are significant imbalances of covariates as discussed in the Descriptive Statistics Section. We select control individuals by matching gender and all the individual and city-level covariates year by year, and apply DID regression using the matched sample.

There may still be the concern that some observable and unobservable factors may miss out, especially those city-level factors that can affect dependent and key independent variables simultaneously. For example, a city with a preferable environment for the elders may accelerate the development of the old-age service industry, which can improve the living conditions and health status of the local elders, and in the meantime, may increase the probability of being selected as the pilot city of LTCI. Using the strategy proposed by Oster [21], we adjust our estimates to assess the influence of omitted observable and unobservable factors.

## Results

### Descriptive statistics

Table 1 shows descriptive statistics for the full sample, the treatment group, and the control group respectively, and the results of pairwise comparisons between the treatment group and the control group before the implementation of LTCI. The mean values of the number of painful body parts, self-reported health score, and depression index of the treatment group are all lower than those of the control group both before and after the pilot. After the pilot, the differences between the two groups on the number of painful body parts and self-reported health score become much larger in magnitude because the increase in the number of painful body parts of the treatment group is much smaller than that of the control group, and the self-reported health score of the control group increases after the pilot while that of the treatment group even decreases, indicating that the treatment group performs better in controlling body pain and improving self-reported health. On the contrary, the growth of depression index of the treatment group is slightly higher than that of the control group, making the difference between the two groups on depression index after the pilot becomes a bit smaller. It seems that the treatment group didn’t work as expected in controlling the deterioration of depression.

**Table 1:** Descriptive Statistics

Variables	Full		Post = 0		Post = 1	
			Treatment	Control	Treatment	Control
	Mean/%	Sd.	Mean/%	Mean/%	Mean/%	Mean/%
Body pain	2.436	3.712	1.854	1.863	2.883	3.582
Self-reported health	3.133	0.960	3.080	3.135	3.019	3.151
Depression	9.492	6.621	8.984	9.445	9.290	9.696
Age	64.09	9.278	63.612	63.370	65.470	65.396
Married and living with spouse = 1	1.043	0.203	1.052*	1.039	1.046	1.049
Education years	4.501	3.893	4.385	4.413	4.416	4.696
Urban residence = 1	0.173	0.378	0.211**	0.180	0.162	0.154
Number of living children	3.058	1.468	2.784***	3.108	2.779	3.039
Natural growth rate of population	6.469	4.676	5.167***	6.908	4.959	6.067
GDP per capita	42.98	27.59	57.497***	36.961	72.749	48.210
Fiscal expenditure per capita	8.092	5.113	10.056***	6.668	14.246	9.712
Number of hospital beds per 1000 inhabitants	4.248	1.527	5.020***	4.088	5.582	4.250
Number of doctors per 1000 inhabitants	2.028	0.899	2.365***	1.835	2.856	2.234

Notes: Sd. Standard deviation. The t-test is applied for pairwise comparisons between the treatment and control groups before the event. ***, **, and * means the significance levels of 1%, 5%, and 10% respectively

We choose covariates from two aspects of individual level and city level to account for possible confounding factors. While individual characteristics contain age, marital status (married and living with spouse = 1), education years, urban residence, and number of living children, the city-level variables include the natural growth rate of population, GDP per capita, fiscal expenditure per capita, number of hospital beds per 1000 inhabitants, and number of doctors per 1000 inhabitants. The results of t-tests demonstrate significant differences between the treatment and control groups on three out of five individual covariates and all five city-level variables, implying that the two groups were unbalanced before the pilot, which needs to be addressed further.

### Baseline estimates

Table 2 reports the estimated effects of LTCI on body pain, self-reported health, and depression. Each column shows the results of a separate regression of Eq. (1) with or without city-level covariates as specified in the table, and the coefficients of interaction Treat×Post are the estimated effects of LTCI on the health outcomes of spouses of disabled individuals. The estimates in columns 1 to 4 demonstrate that LTCI has reduced the number of painful body parts and the self-reported health score significantly, whether city-level covariates are controlled for or not, implying that the implementation of LTCI in pilot cities has improved the physical health status of spouses of disabled individuals. Specifically, the introduction of LTCI is related to a 0.921 decrease in the number of painful body parts and a 0.123 reduction in the self-reported health score when two-way FE and all covariates are controlled for. However, the coefficients of interaction Treat×Post in columns 5 and 6 are not statistically significant, indicating that there is no evidence to support the psychological health effect of LTCI on spouses of disabled individuals.

**Table 2:** Health effects of LTCI on spouses of disabled people

Variables	(1)	(2)	(3)	(4)	(5)	(6)
	Number of painful body parts	Number of painful body parts	Self-reported health score	Self-reported health score	Depression index	Depression index
Treat×Post	-0.898***	-0.921***	-0.140***	-0.123**	-0.162	-0.331
	(0.246)	(0.256)	(0.0466)	(0.0482)	(0.296)	(0.314)
Individual covariates	YES	YES	YES	YES	YES	YES
City-level covariates	NO	YES	NO	YES	NO	YES
Individual FE	YES	YES	YES	YES	YES	YES
Year FE	YES	YES	YES	YES	YES	YES
Observations	12,671	11,440	12,092	10,924	11,799	10,664
R-squared	0.127	0.130	0.009	0.010	0.009	0.010

Notes: Standard errors clustered at the city level are reported in parentheses. ***, **, and * means the significance levels of 1%, 5%, and 10% respectively. Individual covariates include age, marital status, education years, urban residence, and number of living children. City-level variables include the natural growth rate of population, GDP per capita, fiscal expenditure per capita, number of hospital beds per 1000 inhabitants, and number of doctors per 1000 inhabitants

### Robustness

**Fig. 2:**
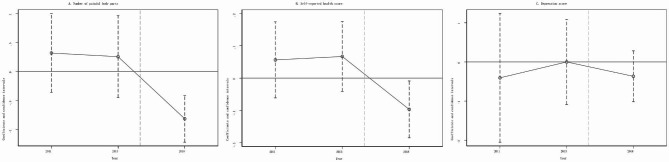
Event study for the effects of LTCI on three health outcomes. Notes: The LTCI was implemented in 2016 or 2017 in the 11 pilot cities. On the x-axis, the year 2015 is omitted because it is treated as the benchmark year. Other Notes are the same as specified in Table 2

First, testing common trends between the treatment and control groups. The common trend assumption of DID specification requires that the trends of health outcomes in both the treatment and control groups are parallel to each other if LTCI had not been implemented. However, the counterfactual trends of outcomes in the treatment group cannot actually be observed, making the event study a prevailing practice to infer the afterward trends of outcomes by examining the trends before the event. Figure 2 shows the estimates of *βt* and their corresponding confidence intervals in Eq. (2). As expected, all coefficients of *β*_*2011*_ and *β*_*2013*_ are insignificant in all three panels, suggesting that there were no pre-intervention differential trends. In addition, consistent with the estimates in Table 2, the estimates of *β*_*2018*_ demonstrate that LTCI reduces the number of painful body parts and self-reported health score significantly but has not a significant impact on the depression index.

Second, combining PSM with DID. PSM is applied to achieve balance on all the observables between the treatment and control groups as there are significant differences between the two groups on many covariates. We first select a more similar sample of individuals in the control group year by year to match those in the treatment group, using the radius matching method within 0.06 calipers of propensity scores estimated by a logit model considering gender and all the individual and city-level covariates in Eq. (1). Then we combine all the matched observations in the four years to obtain a matched sample, containing 9667 observations of 4410 respondents. Finally, the DID regressions are conducted using the matched sample. After matching, the standardized percentage biases of all the covariates are not larger than 10% except for urban residence (-11.2%) in 2011, indicating that there are no significant differences in all the observable characteristics between the treatment group and the control group. The detailed results of balance tests are in Supplementary Table 2A-2D and Supplementary Fig. 1. Table 3 represents the results of PSM-DID. The coefficients of Treat×Post are consistent with baseline estimates both in significance and magnitude, indicating that the baseline estimates are robust.

**Table 3:** Robustness test: PSM-DID

Variables	(1)	(2)	(3)	(4)	(5)	(6)
	Number of painful body parts	Number of painful body parts	Self-reported health score	Self-reported health score	Depression index	Depression index
Treat×Post	-0.916***	-0.906***	-0.122**	-0.123**	-0.341	-0.356
	(0.222)	(0.227)	(0.0541)	(0.0548)	(0.411)	(0.415)
Individual covariates	YES	YES	YES	YES	YES	YES
City-level covariates	NO	YES	NO	YES	NO	YES
Individual FE	YES	YES	YES	YES	YES	YES
Year FE	YES	YES	YES	YES	YES	YES
Observations	9666	9666	9241	9241	9013	9013
R-squared	0.121	0.123	0.007	0.007	0.011	0.011

The Notes are the same as specified in Table 2

Third, selecting the second round of pilot cities as the control group. In 2020, the Chinese government expanded the pilot of LTCI further and added 14 cities/districts to the list of pilot cities, including Shijingshan district in Beijing, Tianjin, etc., which may be more similar to the first ones because it is likely that they are selected to implement LTCI 4 years later depending on the same city-level factors. Therefore, only spouses of disabled individuals in 7 new pilot cities (CHARLS only covers 7 of the 14 pilot cities) remain as the new control group, yielding a selected sample of only 1,594 observations of 702 respondents. The estimates of Eq. (1) using the selected sample are shown in Table 4. Compared to baseline estimates, the estimated effect of LTCI on the number of painful body parts is much stronger in magnitude, and the one on self-reported health score is consistent with that in Table 2, indicating that the significant negative effects of LTCI on physical health status are robust. Surprisingly, the results in columns 5 and 6 show that LTCI also has a significant impact on the depression index, which is not in line with the baseline estimates. This may be due to the short history of LTCI in China, and we expect that the psychological health effects of LTCI will arise in the long run.

**Table 4:** Robustness test: selecting the second round of pilot cities as the control group

Variables	(1)	(2)	(3)	(4)	(5)	(6)
	Number of painful body parts	Number of painful body parts	Self-reported health score	Self-reported health score	Depression index	Depression index
Treat×Post	-1.344**	-1.393**	-0.120*	-0.125**	-1.643**	-1.577**
	(0.574)	(0.610)	(0.0572)	(0.0590)	(0.688)	(0.652)
Individual covariates	YES	YES	YES	YES	YES	YES
City-level covariates	NO	YES	NO	YES	NO	YES
Individual FE	YES	YES	YES	YES	YES	YES
Year FE	YES	YES	YES	YES	YES	YES
Observations	1594	1594	1518	1518	1472	1472
R-squared	0.104	0.113	0.009	0.011	0.024	0.034

The Notes are the same as specified in Table 2

Fourth, controlling for city FE instead of individual FE. Table 5 reports the results of controlling for city FE instead of individual FE, with or without city-level covariates as specified in the table. Although the estimated coefficients of interaction Treat×Post are all slightly lower in magnitude except for the one in column 6, the conclusions remain consistent, that is, LTCI improves the physical health status of spouses of disabled individuals but has no significant impact on their psychological health.

**Table 5:** Robustness test: controlling for city FE instead of individual FE

Variables	(1)	(2)	(3)	(4)	(5)	(6)
	Number of painful body parts	Number of painful body parts	Self-reported health score	Self-reported health score	Depression index	Depression index
Treat×Post	-0.737**	-0.749**	-0.108**	-0.112**	-0.143	-0.389
	(0.292)	(0.296)	(0.0518)	(0.0535)	(0.391)	(0.406)
Individual covariates	YES	YES	YES	YES	YES	YES
City-level covariates	NO	YES	NO	YES	NO	YES
Individual FE	NO	NO	NO	NO	NO	NO
City FE	YES	YES	YES	YES	YES	YES
Year FE	YES	YES	YES	YES	YES	YES
Observations	12,671	11,440	12,092	10,924	11,799	10,664
R-squared	0.144	0.138	0.061	0.058	0.094	0.094

The Notes are the same as specified in Table 2

Fifth, evaluating selection bias from omitted observable and unobservable factors. Until now, we have attempted to control for observable factors at both the individual and city levels to reduce biases from the selectivity of pilot cities and omitted factors. However, there may still be some omitted observable and unobservable factors such as climate, culture, etc., which are associated with the selection into the list of pilot cities and the subsequent health outcomes of residents. We use the adjustment strategy proposed by Oster [21] to test the stability of our estimated coefficients. Using the estimates of baseline as “full controls” specification, we rerun the DID regression considering only individual and year fixed effect as the “restricted controls” specification. Then we adjust the coefficients under the assumptions that R_max_ equals 1.3R^2^, where R^2^ is the R-squared of the baseline regressions, and the relative degree of selection on observables and unobservables δ equals 1.

Table 6 shows the results of the adjustment methodology. While the coefficients of Treat×Post with full controls are reported in column 1, and those with restricted controls model in column 2, the bias-adjusted coefficients are in column 3. After adjustment, all point estimates in column 3 remain negative and quantitatively very close to the estimates in the above models, suggesting that the selectivity of omitted observable and unobservable factors would not affect the stability of our estimates.

**Table 6:** Robustness test: evaluating selection bias from omitted observable and unobservable factors

	(1)	(2)	(3)
	Full controls	Restricted controls	Bias adjusted coefficients
Number of painful body parts			-1.186
Treat×Post	-0.921***	-0.898***	
Std. Err.	(0.256)	(0.246)	
R^2^	0.130	0.127	
Self-reported health score			-0.079
Treat×Post	-0.123**	-0.140***	
Std. Err.	(0.0482)	(0.0466)	
R^2^	0.010	0.009	
Depression index			-0.631
Treat×Post	-0.331	-0.162	
Std. Err.	(0.314)	(0.296)	
R^2^	0.010	0.009	

Notes: Full controls include individual covariates and city-level covariates as specified in Table 2, and also individual FE and year FE, while restricted controls only contain individual FE and year FE. Standard errors clustered at the city level are reported in parentheses. ***, **, and * means the significance levels of 1%, 5%, and 10% respectively

### Channels

Aiming to relieve the financial and care burdens of households with disabled individuals, the introduction of LTCI could exert health effects on spouses of disabled people through two channels: time reallocation and consumption promotion. Households with disabled people may increase their consumption of food, fuels, etc. because the current and expected financial burdens related to LTC will decrease with the coverage of LTCI, from which spouses can gain health benefits. Meanwhile, as the primary family caregivers, spouses may save time from the decrease of care burden and devote it to health production such as sleeping, doing physical exercises, and participating in social activities, etc., which can also improve their health status by increasing their health stock.

Table 7 represents the estimates of the time reallocation and consumption promotion channels. LTCI is negatively related to the probability of spouse caregiving and spouse caregiving hours, although the latter is not significant, indicating that LTCI has reduced the care burden of spouses, especially by making them choose other types of caregiving. Furthermore, we find that LTCI increases spouses’ actual sleep hours at night, but there is little evidence to support the effects of LTCI on doing physical exercises and participating in social activities, suggesting that spouses of disabled people mainly devote the saved time to sleeping, which is demonstrated to be beneficial to health [22]. However, the estimates of LTCI on household expenditure per capita and household survival consumption per capita, which is defined as the yearly expenditure of every household member spending on food, clothing, utilities, fuel, communication, daily necessities, and transportation according to the classification of Huang and Hu [23], in columns 6 and 7 are both insignificant, indicating that spouses of disabled individuals didn’t gain health benefits through the consumption promotion channel.

**Table 7:** Channels of time reallocation and consumption promotion

Variables	(1)	(2)	(3)	(4)	(5)	(6)	(7)
	Caring for disabled spouses	Care-giving hours	Hours of actual sleep	Doing physical exercises	Participating in social activities	Household expenditure per capita	Household survival consumption per capita
Treat×Post	-0.0711*	-0.213	0.0645**	0.0473	0.0399	0.0412	0.0757
	(0.0426)	(0.169)	(0.0285)	(0.0314)	(0.0404)	(0.0646)	(0.0943)
Observations	11,335	8761	10,887	7105	11,440	11,190	11,212
R-squared	0.125	0.081	0.010	0.011	0.009	0.088	0.054

Notes: Each regression controls for individual covariates, city-level covariates, individual FE, and year FE. Other Notes are the same as specified in Table 2

### Heterogeneity

Previous studies reveal that the effects of LTCI on various outcomes such as informal caregiving hours [11], care recipients’ well-being [12], caregivers’ labor force participation [1, 2], and children’s intergenerational transfer to their parents [24], etc. differ across different individual characteristics or policy designs. Therefore, according to each dummy grouping variable, the full sample is divided into two subsamples and DID regressions are conducted as specified in Eq. (1) for each subsample. To compare the estimates between each pair of divided subgroups, a triple-difference estimation is selected by regressing Eq. (1) with added dummy grouping variable (GV) and its interactions with all the covariates using the full sample, and the F test is used to determine the significance of the interaction GV×Treat×Post. The results of group-level regressions and the corresponding F test are reported in Table 8.

**Table 8:** Heterogenous health effects of LTCI on spouses of disabled people

Variables	(1)	(2)	(3)	(4)	(5)	(6)
	Number of painful body parts	Self-reported health score	Depression index	Number of painful body parts	Self-reported health score	Depression index
Panel A	Not caregivers			Caregivers		
Treat×Post	-0.796**	0.0660	-0.168	-0.785*	-0.202*	-0.879
	(0.358)	(0.0571)	(0.502)	(0.461)	(0.102)	(0.666)
Observations	5392	5022	4872	5943	5799	5697
R-squared	0.135	0.020	0.023	0.133	0.008	0.011
F-statistic	0.39	6.24	0.25			
P-value	0.533	0.014	0.621			
Panel B	Lower-level education			Higher-level education		
Treat×Post	-1.457***	-0.191*	-1.118*	-0.146	-0.0660	0.133
	(0.256)	(0.100)	(0.575)	(0.322)	(0.0479)	(0.402)
Observations	5956	5625	5473	5484	5299	5191
R-squared	0.142	0.012	0.015	0.119	0.010	0.010
F-statistic	13.6	0.86	0.44			
P-value	0.001	0.356	0.507			
Panel C	Lower-income			Higher-income		
Treat×Post	-2.375***	-0.240	2.036	-1.031	0.143	0.203
	(0.861)	(0.322)	(1.706)	(0.627)	(0.110)	(0.631)
Observations	2748	2602	2542	2739	2625	2583
R-squared	0.087	0.029	0.009	0.229	0.043	0.016
F-statistic	3.50	0.74	1.41			
P-value	0.064	0.393	0.238			

The Notes are the same as specified in Table 7

In Table 8, panel A, the full sample is divided by the caregiving choice of spouses. All spouses of disabled individuals benefited from the implementation of LTCI, whether they assisted his/her disabled partner or not. However, the overall beneficial health effects are stronger for spouse caregivers. Specifically, for those who were not spouse caregivers, LTCI reduces their number of painful body parts significantly, and hasn’t significant effects on their other two health outcomes; for those who were spouse caregivers, LTCI decreases both their number of painful body parts and self-reported health score, and only hasn’t significant effect on their depression index. Furthermore, the results of F tests in panel A, column 2 demonstrate that the impact of LTCI on the self-reported health score for spouse caregivers is significantly larger than for non-spouse caregivers.

Table 8, panels B and C report the heterogeneous health effects of LTCI on spouses of disabled people across education years and household income per capita respectively. In panel B, the coefficients of Treat×Post are all significant for the lower-level education group, which includes individuals whose education years are not above three years, while by contrast, the estimates for the higher-level education group are all insignificant. Furthermore, the three estimates for the lower-level education group are much larger than those for the higher-level education group, and the results of F tests in panel B, column 1 illustrate that the effect of LTCI on the number of painful body parts for the lower-level education group is significantly larger than for the higher-level education group. In panel C, individuals whose household income per capita is below and equal to the 25th percentile are classified into the lower-income group while those above the 75th percentile fall into the higher-income group. For the lower-income group, LTCI only reduces the number of painful body parts, but hasn’t significant effects on their other two health outcomes, while for the lower-income group, the three estimates are all insignificant. Furthermore, the results of F tests in panel C, column 1 show that the effect of LTCI on the number of painful body parts for the lower-income group is significantly stronger than for the higher-income group. In sum, the beneficial health effects of LTCI are larger for spouses with lower socio-economic status.

## Conclusion and discussion

This quasi-experimental study examines the health effects of LTCI on spouses of disabled people in China, using nationally representative survey data from CHARLS and statistical data from China City Statistical Yearbook. Three findings from DID estimates are as follows. Firstly, the introduction of LTCI in China reduces the number of painful body parts and the self-reported health score significantly, indicating that spouses of disabled people get physical health benefits from LTCI coverage. However, the impact of LTCI on the depression index remains ambiguous and needs to be analyzed further. Secondly, LTCI improves the physical health status of spouses of disabled individuals mainly through the time reallocation channel, while the impact of the consumption promotion channel has not been verified. Thirdly, the beneficial effects of LTCI on physical health are stronger for spouse caregivers, and spouses with lower-level education and lower household income, suggesting that LTCI plays a more important role in helping caregivers or family members with lower socio-economic status cope with the financial and care burdens from LTC.

Our findings of beneficial physical health effects of LTCI add new evidence from a developing country to support the positive health effects of formal LTC supply on caregivers. Previous studies have so far reported inconsistent results. Miyawaki et al. [3] find that reduced LTC benefits caused by Japan’s LTCI reform in 2006 deteriorate the health status of caregivers, and Wagner and Brandt [25] find that regional formal LTC supply is positively associated with the well-being of spouse caregivers in 11 European countries, making them feel more satisfied with life, less depressed and less lonely. However, some other studies find little health effects of daycare on caregivers. Mossello et al. [26] report that although a two-month period of daycare use can alleviate the caregiving burden of caregivers, their depressive symptoms do not change, and Zank and Schacke [27] even argue that they do not find any effects of geriatric daycare on both caregiving burden and subjective well-being.

Concerning the time reallocation channel, we demonstrate that LTCI can significantly reduce the proportion of spouse caregiving by 7.11% points, so spouses can improve their health status by devoting more time to sleep. These findings are in line with that of Cai et al. [24], who find that LTCI in China significantly decreases the possibility of choosing family care by 20.5% points, and Lee et al. [28], who report that institutional respite care is positively related to total sleep time per night and subjective sleep quality of dementia caregivers. Our estimate of 7.11% points is much smaller than that of Cai et al. [24], and one candidate explanation is that there may be more other family caregivers especially children who choose to give up informal care than spouses because of LTCI.

From the perspective of the consumption promotion channel, we find that the estimates of LTCI on household total consumption per capita and household survival consumption per capita are both insignificant, which is inconsistent with the results of Iwamoto et al. [29], who find that the negative effect of LTC cost on household consumption in 1998 no longer existed in 2001 and argue that it is Japan’s introduction of LTCI in 2000 that mitigates the consumption risk caused by the disabled household members, and Ariizumi [30], who find that public means-tested LTC programs are positively related to initial expenditure on consumption goods among the middle-wealth group of people. However, as discussed by Liu et al. [31], the impacts of LTCI on non-health consumption for households with disabled individuals depend on the price elasticity of demand for LTC services. If the price elasticity is less than one, out-of-pocket LTC expenditure will decrease, and households may spend the saved money on more non-health consumption. On the contrary, if the price elasticity is larger than one, out-of-pocket LTC expenditure may increase, and households may reduce the money spent on non-health consumption. For a long time, more than half of the disabled elders had unmet LTC needs in China [32], leading to massive pent-up demands for LTC, which will transform into effective demands after the LTCI coverage. Therefore, in the initial phase of the LTCI pilot this study examines, out-of-pocket LTC expenditure may increase rapidly because the release effect of LTCI on the demand for LTC may hold a dominant position.

The results of heterogeneity analysis show that the overall beneficial health effects are larger for spouses with lower socio-economic status, which is in line with that of Lei et al. [12], who report that LTCI is more effective in improving the well-being of lower-income older disabled people. Our findings complement the existing studies by illustrating that LTCI also benefits more for family members with lower socio-economic status, implying that LTCI can be helpful in reducing health disparities and promoting health equity. Regarding the heterogeneous health effects of LTCI on caregiving and non-caregiving spouses, caregivers may experience greater perceived burdens, worse sleep quality, and more depressive symptoms [33, 34], and can inevitably benefit more from LTCI coverage through the time reallocation channel. Note that both spouse caregivers and non-caregivers get health benefits from LTCI coverage, which is in keeping with that of Bobinac et al. [35], who argue that the well-being effects of healthcare interventions on significant others including spouses, children, etc. contain not only the caregiving effect, which comes from the decrease of care burdens, but also the family effect, which is defined as the direct welfare benefits from caring about the sick family member.

Overall, our findings demonstrate that LTCI can not only improve the health status of family caregivers by reducing their caregiving burden but also have beneficial health effects on non-caregiver family members. Policy designs of LTCI should emphasize the orientation of home and community-based services (HCBS). Currently, only 5 out of 15 pilot cities, namely Shanghai, Suzhou, Jingmen, Guangzhou, and Chengdu, encourage households with disabled individuals to choose HCBS in terms of higher reimbursement rates or/and ceilings, while in other pilot cities, the benefit levels for institutional care services equal or even higher than those for HCBS. However, more than 90% of older people in China prefer to be cared for at home by family members rather than going to a nursing home, according to the results of a survey published by the National Health Commission in 2021. Therefore, policy designs that encourage the use of HCBS not only satisfy the care preferences of disabled individuals, reduce the care burden on family caregivers, and promote the health of all family members, but also prevent a large number of disabled people from choosing high-cost institutional care, which may be conducive to the financial sustainability of the long-term care insurance fund. In this way, we can achieve the maximization of the individual-family-society utility.

There are several potential limitations in this study. Firstly, because there is no information on whether disabled individuals receive or are qualified to receive LTCI benefits, we estimate the intent-to-treat effects of LTCI instead of treatment effects. However, previous studies illustrate that public programs, such as the poverty alleviation program and social pension, can not only affect the enrolled or eligible households directly but also influence other households not enrolled in the programs or ineligible for the benefits in the same region [19, 20], making intent-to-treat effects, not treatment effects, more attractive to policymakers. Secondly, with information on only one wave after the implementation of LTCI, we can only illustrate the short-term health effects, and there is a lack of understanding about the long-term impact of LTCI, which may be a concern in future research.

### Electronic supplementary material

Below is the link to the electronic supplementary material.

Supplementary Material 1

## Data Availability

The datasets used in this study is from China Health and Retirement Longitudinal Survey (CHARLS), which are publicly available in the CHARLS repository. https://charls.pku.edu.cn/en. The datasets used and/or analysed during the current study are available from the corresponding author on reasonable request.
