# Supplementary material for: Health effects of long-term care insurance on spouses of disabled people: a quasi-experimental study
Source: BMC Geriatr. 2023 Oct 19;23:679. doi: 10.1186/s12877-023-04344-9 (PMC10588235; doi:10.1186/s12877-023-04344-9)
Supplement: Supplementary file 1 — Supplementary Material 1 [file 12877_2023_4344_MOESM1_ESM.docx]

### Supplementary Section 1：Key policy issues of LTCI programs in 15 pilot cities

### Supplementary Table 1

### Key policy issues of LTCI programs in 15 pilot cities

| Key policy issues | Classifications | Pilot cities |
| --- | --- | --- |
| Coverage | Urban employees enrolled in UEBMI | Chengde, Qiqihaer, Ningbo, Anqing, Shangrao, Guangzhou, Chongqing, Chengdu |
|  | Urban employees enrolled in UEBMI + urban residents enrolled in URBMI | Changchun, Nantong |
|  | Urban employees enrolled in UEBMI + urban and rural residents enrolled in URRBMI | Suzhou, Shanghai, Jingmen, Shihezi, Qingdao |
| Funding channels | Medical insurance fund | Changchun (Urban residents), Ningbo, Guangzhou, Qingdao |
|  | Medical insurance fund+ individual contributions | Changchun (Urban employees), Qiqihaer, Anqing, Chongqing, |
|  | Medical insurance fund+ Employer contribution/ Government subsidies | Shanghai |
|  | Medical insurance fund+ individual contributions+ Employer contribution | Chengde, Suzhou, Nantong, Jingmen, Shihezi, Chengdu |
|  | Medical insurance fund+ individual contributions + Employer contribution+ Government subsidies | Shangrao |
| Eligibility criteria | Disabled people with severe disability | Chengde, Qiqihaer, Ningbo, Anqing, Jingmen, Chongqing, Chengdu, Shihezi |
|  | Disabled people with severe, moderate, mild disability or severe dementia | Changchun, Shanghai, Suzhou, Nantong, Shangrao, Guangzhou, Qingdao |
| Benefit forms | Only formal care | Chengde, Qiqihaer, Shanghai, Suzhou, Ningbo, Anqing, Guangzhou, Chongqing, Qingdao, Changchun, Ningbo |
|  | Both formal care and cash allowance | Nantong, Shangrao, Jingmen, Chengdu, Shihezi |
| Reimbursement ceilings | Above 100 RMB/person/day | Changchun, Shanghai, Jingmen, Guangzhou, Qingdao |
|  | Below 60 RMB/person/day | Chengde, Qiqihaer, Suzhou, Nantong, Ningbo, Anqing, Shangrao, Chongqing, Chengdu, Shihezi |

### Supplementary Section 2：Balance test results of PSM

### Supplementary Table 2A

### Balance test results of characteristic variables in 2011

| Variable | Unmatched | Mean | | %bias | %reduct | t-test | |
| --- | --- | --- | --- | --- | --- | --- | --- |
|  | Matched | Treated | Control |  | \|bias\| | t | p>\|t\| |
| Male | U | 0.602 | 0.556 | 9.300 |  | 0.920 | 0.358 |
|  | M | 0.604 | 0.585 | 3.900 | 58.40 | 0.280 | 0.778 |
| Age | U | 62.07 | 62.03 | 0.400 |  | 0.0400 | 0.965 |
|  | M | 61.91 | 62.45 | -6.100 | -1265 | -0.440 | 0.663 |
| Married and living together | U | 1.074 | 1.043 | 13 |  | 1.460 | 0.145 |
|  | M | 1.075 | 1.085 | -3.900 | 69.80 | -0.250 | 0.804 |
| Education years | U | 4.556 | 4.296 | 6.900 |  | 0.670 | 0.503 |
|  | M | 4.500 | 4.620 | -3.200 | 53.60 | -0.230 | 0.815 |
| Urban residence | U | 0.213 | 0.186 | 6.600 |  | 0.670 | 0.501 |
|  | M | 0.198 | 0.243 | -11.20 | -70.10 | -0.790 | 0.431 |
| Number of living children | U | 2.685 | 3.110 | -28.30 |  | -2.860 | 0.00400 |
|  | M | 2.708 | 2.807 | -6.600 | 76.70 | -0.480 | 0.631 |
| Natural growth rate of population | U | 5.900 | 5.899 | 0 |  | 0 | 0.997 |
|  | M | 6.024 | 5.994 | 0.900 | -2286 | 0.0600 | 0.950 |
| Ln(GDP per capita) | U | 10.49 | 10.18 | 58.30 |  | 5.960 | 0 |
|  | M | 10.48 | 10.53 | -8.600 | 85.30 | -0.560 | 0.577 |
| Ln (Fiscal expenditure per capita) | U | 8.735 | 8.327 | 77.40 |  | 8.970 | 0 |
|  | M | 8.707 | 8.699 | 1.500 | 98.10 | 0.100 | 0.922 |
| Number of hospital beds per 1000 inhabitants  r | U | 3.921 | 3.339 | 40.50 |  | 4.540 | 0 |
|  | M | 3.854 | 3.822 | 2.300 | 94.40 | 0.160 | 0.870 |
| Number of doctors per 1000 inhabitants  r | U | 1.985 | 1.663 | 39.30 | 4.320 | 0 | 1.57* |
|  | M | 1.965 | 1.992 | -3.200 | 91.80 | -0.230 | 0.821 |

Notes: We use 1 to 5 nearest neighbors matching within 0.2 calipers of propensity scores, which are estimated by a logit model considering gender, education years, age, age’s square, marital status, urban residence, number of living children, number of household members, ln(GDP per capita), ln(fiscal expenditure per capita), natural growth rate of population, number of hospital beds per 1000 inhabitants and number of doctors per 1000 inhabitants.

### Supplementary Table 2B

### Balance test results of observable variables in 2013

| Variable | Unmatched | Mean | | %bias | %reduct | t-test | |
| --- | --- | --- | --- | --- | --- | --- | --- |
|  | Matched | Treated | Control |  | \|bias\| | t | p>\|t\| |
| Male | U | 0.597 | 0.578 | 3.900 |  | 0.570 | 0.568 |
|  | M | 0.592 | 0.587 | 0.900 | 76.90 | 0.100 | 0.922 |
| Age | U | 64.06 | 63.20 | 9.700 |  | 1.420 | 0.157 |
|  | M | 64.03 | 64.16 | -1.500 | 84.70 | -0.160 | 0.870 |
| Married and living together | U | 1.054 | 1.033 | 10 |  | 1.640 | 0.102 |
|  | M | 1.050 | 1.044 | 3.200 | 67.80 | 0.340 | 0.735 |
| Education years | U | 4.251 | 4.450 | -5.300 |  | -0.770 | 0.444 |
|  | M | 4.254 | 4.285 | -0.800 | 84.40 | -0.0900 | 0.925 |
| Urban residence | U | 0.218 | 0.184 | 8.400 |  | 1.280 | 0.199 |
|  | M | 0.221 | 0.239 | -4.500 | 46.90 | -0.470 | 0.641 |
| Number of living children | U | 2.946 | 3.108 | -11.30 |  | -1.650 | 0.0980 |
|  | M | 2.954 | 2.918 | 2.600 | 77.50 | 0.280 | 0.781 |
| Natural growth rate of population | U | 4.516 | 6.991 | -66.30 |  | -7.780 | 0 |
|  | M | 4.563 | 4.647 | -2.300 | 96.60 | -0.320 | 0.752 |
| Ln(GDP per capita) | U | 10.68 | 10.29 | 61.10 |  | 9.740 | 0 |
|  | M | 10.66 | 10.60 | 9.300 | 84.70 | 0.940 | 0.350 |
| Ln (Fiscal expenditure per capita) | U | 8.990 | 8.639 | 71.90 |  | 11.62 | 0 |
|  | M | 8.976 | 8.941 | 7.100 | 90.10 | 0.710 | 0.481 |
| Number of hospital beds per 1000 inhabitants  r | U | 4.888 | 3.933 | 58.80 |  | 10.58 | 0 |
|  | M | 4.852 | 4.727 | 7.700 | 87 | 0.780 | 0.438 |
| Number of doctors per 1000 inhabitants  r | U | 2.351 | 1.821 | 55.90 |  | 9.370 | 0 |
|  | M | 2.338 | 2.243 | 10 | 82.20 | 1 | 0.320 |

Notes: The same as specified in Table 1A.

### Supplementary Table 2C

### Balance test results of observable variables in 2015

| Variable | Unmatched | Mean | | %bias | %reduct | t-test | |
| --- | --- | --- | --- | --- | --- | --- | --- |
|  | Matched | Treated | Control |  | \|bias\| | t | p>\|t\| |
| Male | U | 0.593 | 0.591 | 0.400 |  | 0.0700 | 0.944 |
|  | M | 0.589 | 0.583 | 1.100 | -181.8 | 0.140 | 0.885 |
| Age | U | 63.77 | 64.24 | -5 |  | -0.900 | 0.366 |
|  | M | 63.69 | 63.68 | 0.100 | 97.30 | 0.0200 | 0.985 |
| Married and living together | U | 1.044 | 1.040 | 2.200 |  | 0.400 | 0.692 |
|  | M | 1.039 | 1.047 | -3.700 | -69.70 | -0.480 | 0.630 |
| Education years | U | 4.424 | 4.581 | -4.200 |  | -0.730 | 0.467 |
|  | M | 4.448 | 4.522 | -2 | 52.80 | -0.270 | 0.790 |
| Urban residence | U | 0.205 | 0.173 | 8.100 |  | 1.500 | 0.133 |
|  | M | 0.203 | 0.219 | -4 | 50.70 | -0.510 | 0.609 |
| Number of living children | U | 2.704 | 3.080 | -25.80 |  | -4.570 | 0 |
|  | M | 2.721 | 2.760 | -2.700 | 89.70 | -0.360 | 0.719 |
| Natural growth rate of population | U | 5.386 | 7.207 | -39.90 |  | -6.010 | 0 |
|  | M | 5.474 | 5.362 | 2.400 | 93.90 | 0.360 | 0.719 |
| Ln(GDP per capita) | U | 10.82 | 10.49 | 61.60 |  | 11.71 | 0 |
|  | M | 10.80 | 10.76 | 8.200 | 86.60 | 1.040 | 0.299 |
| Ln (Fiscal expenditure per capita) | U | 9.209 | 8.848 | 83.30 |  | 16.05 | 0 |
|  | M | 9.193 | 9.161 | 7.400 | 91.10 | 0.890 | 0.371 |
| Number of hospital beds per 1000 inhabitants  r | U | 5.437 | 4.456 | 54.90 |  | 12.05 | 0 |
|  | M | 5.386 | 5.226 | 9 | 83.60 | 1.140 | 0.257 |
| Number of doctors per 1000 inhabitants  r | U | 2.487 | 1.905 | 56.40 |  | 12.44 | 0 |
|  | M | 2.467 | 2.374 | 9.100 | 83.90 | 1.140 | 0.255 |

Notes: The same as specified in Table 1A.

### Supplementary Table 2D

### Balance test results of observable variables in 2018

| Variable | Unmatched | Mean | | %bias | %reduct | t-test | |
| --- | --- | --- | --- | --- | --- | --- | --- |
|  | Matched | Treated | Control |  | \|bias\| | t | p>\|t\| |
| Male | U | 0.579 | 0.590 | -2.200 | -0.420 | 0.676 | . |
|  | M | 0.569 | 0.579 | -2 | 8.300 | -0.280 | 0.779 |
| Age | U | 65.47 | 65.60 | -1.400 | -0.260 | 0.796 | 0.940 |
|  | M | 65.44 | 65.18 | 2.900 | -106.5 | 0.400 | 0.691 |
| Married and living together | U | 1.046 | 1.049 | -1.600 | -0.300 | 0.763 | 0.940 |
|  | M | 1.044 | 1.054 | -4.700 | -189.4 | -0.640 | 0.522 |
| Education years | U | 4.416 | 4.745 | -8.800 | -1.590 | 0.111 | 0.830 |
|  | M | 4.434 | 4.524 | -2.400 | 72.40 | -0.340 | 0.732 |
| Urban residence | U | 0.162 | 0.150 | 3.400 | 0.660 | 0.510 | . |
|  | M | 0.166 | 0.175 | -2.300 | 33.90 | -0.310 | 0.760 |
| Number of living children | U | 2.779 | 3.041 | -18.30 | -3.520 | 0 | 1.090 |
|  | M | 2.810 | 2.779 | 2.200 | 87.90 | 0.310 | 0.753 |
| Natural growth rate of population | U | 4.959 | 6.104 | -28.30 | -5.460 | 0 | 1.120 |
|  | M | 5.022 | 5.222 | -4.900 | 82.60 | -0.670 | 0.502 |
| Ln(GDP per capita) | U | 11.01 | 10.65 | 67 | 13.17 | 0 | 1.23* |
|  | M | 10.99 | 10.98 | 1.100 | 98.40 | 0.140 | 0.888 |
| Ln (Fiscal expenditure per capita) | U | 9.426 | 9.092 | 82 | 16.43 | 0 | 1.34* |
|  | M | 9.407 | 9.367 | 9.900 | 87.90 | 1.300 | 0.193 |
| Number of hospital beds per 1000 inhabitants  r | U | 5.582 | 4.245 | 71.10 | 16.21 | 0 | 2.31* |
|  | M | 5.517 | 5.479 | 2 | 97.20 | 0.250 | 0.803 |
| Number of doctors per 1000 inhabitants  r | U | 2.856 | 2.235 | 58.10 | 12.74 | 0 | 1.97* |
|  | M | 2.812 | 2.747 | 6.100 | 89.60 | 0.770 | 0.442 |

Notes: The same as specified in Table 1A.

### Supplementary Figure 1

### Density figures before and after PSM

Notes: The matched sample is combined by all the matched observations in the four years.
